# Band Gaps of Hexagonal ScN and YN Multilayer Materials

**DOI:** 10.3390/ma18132938

**Published:** 2025-06-21

**Authors:** Maciej J. Winiarski

**Affiliations:** Institute of Low Temperature and Structure Research, Polish Academy of Sciences, Okólna 2, 50-422 Wrocław, Poland; m.winiarski@intibs.pl

**Keywords:** nitride semiconductors, band gap, calculations

## Abstract

The structural parameters and electronic structures of Sc- and Y-based nitride semiconductors that adopted hexagonal BN-like atomic sheets were investigated with calculations based on density functional theory (DFT). A hybrid exchange-correlation functional and spin–orbit coupling were employed for studies on the band structures. A strong variation in the band gap type, as well as the width, was revealed not only between the monolayer and bulk materials but also between the multilayer systems. An exceptionally wide range of band gaps from 1.39 (bulk) up to 3.59 eV (three layers) was obtained for two-dimensional materials based on ScN. This finding is related to two phenomena: significant contributions of subsurface ions into bands that formed a valence band maximum and pronounced shifts in conduction band positions with respect to the Fermi energy between the multilayer systems. The relatively low values of the work function (below 2.36 eV) predicted for the few-layer YN materials might be considered for applications in electron emission. In spite of the fact that the band gaps of two-dimensional materials predicted with hybrid DFT calculations may be overestimated to some extent, the electronic structure of homo- and heterostructures formed by rare earth nitride semiconductors seems to be an interesting subject for further experimental research.

## 1. Introduction

Two-dimensional (2D) boron nitride (hBN) draws wide-ranging interest because of its potential applications in photonics [1]. A possible formation of diverse van der Waals heterostructures based on hBN is a promising area for further experimental and theoretical research. Other group III nitride semiconductors adopt the wurtzite structure, which strongly differs from the graphene-like atomic sheets present in hBN. Among transition metal nitride compounds, ScN is expected to form a metastable phase of the hBN type [2]. Experimental studies on the crystal structure of Sc-doped GaN materials supported this prediction [3]. The atomic layers containing Sc ions are strongly flattened with respect to the 3D wurtzite structure of a host material. Recent investigations that employed density functional theory (DFT) indicate that the ground-state structure of the rock salt type in bulk rare earth (*RE*) nitrides (ScN, YN, LuN) becomes unstable under tensile strain [4,5]. In such conditions the 2D hBN phase is the most energetically favorable, which results in the strongly decreased *c/a* ratios found in *RE*-doped group III (Al, Ga, In) nitride semiconductors [6].

The structural and electronic properties of a hexagonal monolayer of ScN were extensively studied in recent years [7,8,9]. Two-dimensional atomic sheets of hScN may exhibit promising thermoelectric performance [10]. Hydro-fluorinated monolayers are an example of a possible direction for surface engineering in hScN-based systems [11]. The presence of local magnetic moments related to the Sc and N vacancies or acceptor impurities (alkali metals) was recently predicted for monolayer ScN [12]. An Y-based nitride monolayer is also expected to be thermodynamically stable according to theoretical predictions [13].

Although the ranges of indirect band gaps ($E_{g}^{\Gamma -X}$ and $E_{g}^{\Gamma -K}$) in the rock-salt- (0.9–1.3 eV [14,15,16,17,18]) and hBN-type (1.34–1.69 eV [19]) *RE*N semiconductors are rather narrow, a relatively wide range of $E_{g}^{\Gamma -M}$ (1.12–2.32 eV [8,19]) was predicted for the corresponding monolayer systems. Strong spin–orbit coupling (SOC) in these materials leads to interesting features of their electronic structure. A comparable in value $E_{g}$ and similar SOC strength are characteristic of 2D transition metal dichalcogenide materials [20].

In this work, the structural and electronic properties of homolayer ScN and YN systems were investigated with DFT calculations. The band structures were obtained within the fully relativistic approach due to a considerable influence of SOC on the electronic structure of *RE* nitrides [19]. The hybrid exchange-correlation functional (HSE [21]) was used for the calculations of the band gaps and band offsets. The discussion is focused mainly on an evolution of the band structures among the multilayer systems, which was surprisingly complex in the hexagonal ScN and YN materials.

## 2. Materials and Methods

The VASP package [22,23] was used for the DFT-based calculations. The plane-wave-augmented (PAW [24]) atomic datasets, including the semi-core states, were employed. The 500 eV plane-wave energy cutoff was set. The Perdew–Burke–Ernzerhof (GGA [25]) and hybrid Heyd–Scuseria–Ernzerhof (HSE [21]) parametrizations were selected for the exchange-correlation functional. The multilayer systems were modeled with symmetric slabs containing a vacuum region bigger than 12 Å. All structural parameters were fully relaxed via stresses/forces optimization with the GGA functional and van der Waals correction D3 [26,27]. Next, the band structures were calculated within the HSE approach with spin–orbit coupling included. The **k**-point meshes of 8 × 8 × 6 and 8 × 8 × 4 were selected for bulk and slab systems, respectively. The standard **k**-point lattice for slab calculations with 1 division in the Z direction was insufficient due to abnormal band shapes related to the fully relativistic exact-exchange operator in the VASP package.

## 3. Results and Discussion

The structural parameters calculated for the hexagonal phases of the ScN and YN are gathered in Table 1. As one may expect, the GGA-derived lattice parameters of the studied systems were bigger than the previous LDA predictions. The *c*/*a* ratio did not depend on the selection of the exchange-correlation functional. The in-plane lattice parameters of the hScN and hYN were relatively big compared with the case of hBN (2.50 Å [28]). A comparable *a* was characteristic of MoTe_2_ (3.52 Å [29]), whereas the InSe monolayer exhibited a bigger *a* (4.00 Å [30]). One might consider some van der Waals heterostructures formed from *RE*N and TMDC materials, similarly to the recent development of systems based on hBN [31]. It is also worth recalling that the hexagonal phase of LuN is expected to exhibit a smaller unit cell than that of hYN [19]. The van der Waals correction at the D2 level causes a strong decrease in the unit cell volume and an slight increase in the *c*/*a* ratio of the materials. Interestingly, the effect of the D3 correction on the structural parameters of hexagonal *RE* nitrides was balanced and led to crystal structures similar to those obtained from the LDA calculations. The findings presented here indicate a rather small contribution of the van der Waals force into overall bonding in *RE* nitride compounds. The electronic structure of $RE^{3+}$ ions with open *d* shells was clearly different from that of B^3+^ ions in the hBN. Despite the fact that the influence of the van der Waals correction on the structural parameters of bulk materials was not very strong, it may be significant in some multilayer systems. The electronic structure results discussed in this work were obtained with the D3 correction included.

A relative reduction in the lattice parameter *a* was expected in the *RE*N monolayer (1L) systems. As presented in Table 1, the lattice parameter *a* of the 1L ScN was smaller by ≈0.2 Å with respect to the in-plane lattice parameter of the bulk material. A similar effect was pronounced in the case of an Y-bearing nitride. The bigger value of *a* reported for the 1L YN in the literature is related to the repulsive Hubbard *U* term in the GGA+*U* approach.

**Figure 1:**
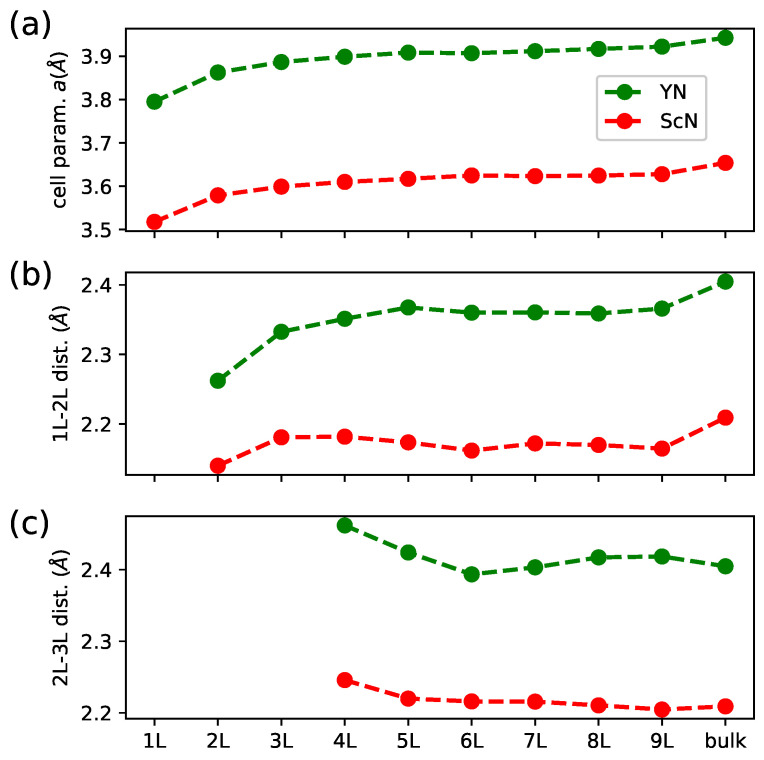
The in-plane lattice parameters *a* (**a**) and the distances between the first and second (1L–2L) (**b**) and the second and third (2L–3L) atomic layers (**c**) calculated (GGA+D3) for the multilayer ScN and YN materials.

The dependence of *a* on a number of atomic layers in multilayer ScN and YN materials is depicted in Figure 1a. The strongly reduced *a* with respect to the bulk, which was obtained for the 1L materials, increased with an increasing number of atomic layers in slab systems. The most pronounced change in *a* was found between the 1L and 2L cases, whereas the variation in *a* for the 5L–9L cases was almost negligible at the scale of this figure. It is worth noting that the relaxed crystal structures of the relatively big 9L slabs incompletely resembled those of the bulk compounds, although the influence of surface ions on the equilibrium *a* of a material became very small in these cases. The careful insight into slab relaxations may be based on the evolution of an interlayer distance in systems that are bigger than a monolayer. As presented in Figure 1b, the out-of-plane lattice parameter was strongly decreased in the bilayers, whereas the variation in the 1L–2L distance between the 3L–9L slabs was relatively small. The structural relaxation in the out-of-plane direction may be difficult in Y-bearing systems due to a big ionic radius of this element. One may notice that the 1L–2L distance in the bilayer YN was only slightly bigger than that of the bulk ScN compound. It is worth recalling that the presence of relatively big ions of transition metal elements in dichalcogenides is balanced with some additional structural parameters (the free parameter *u* [20]), which results in a less 2D character of the monolayers with well-relaxed bonding. Furthermore, some nitride systems adopt more complex and anisotropic sandwich-like 2D structures [32]. The relaxation of the second interlayer distance, as depicted in Figure 1c, resulted in the bulk-like equilibrium structure in slabs bigger than 5L and 6L for the ScN and YN, respectively. This finding indicates the limitation of changes in bonding related to charge imbalance at the surface of hexagonal *RE* nitrides to the nearest subsurface atomic layers.

**Figure 2:**
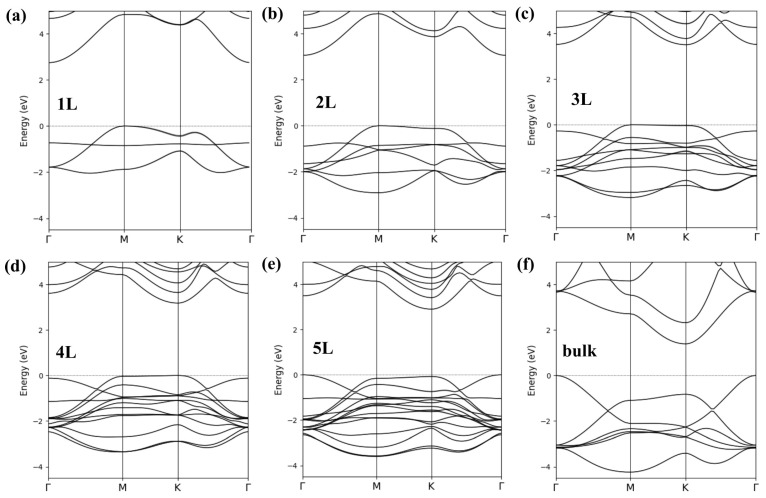
Band structures calculated within the HSE approach for 1L (**a**), 2L (**b**) 3L (**c**), 4L (**d**), 5L (**e**), and bulk (**f**) hexagonal ScN systems.

The band structure for the 1L ScN depicted in Figure 2a reveals the valence band maximum (VBM) at the M point and conduction band minimum (CBM) located at the Γ point in the Brillouin zone. The indirect $E_{g}^{M-\Gamma }$ of 2.74 eV obtained with the HSE functional is wider than the previous results of the full-potential calculations within the modified Becke–Johnson approach (2.2–2.3 eV [8,19]). A similar issue was also reported for other 2D nitride materials [33] and may be tuned in some extent with an adjustment of the mixing parameter α (the exact exchange contribution) in the HSE functional [34]. The origin of this discrepancy is not the PAW atomic datasets because the $E_{g}^{K-\Gamma }$ of the bulk ScN compound (1.39 eV) is close to the MBJGGA-derived one (1.34 eV [19]). The overall shapes of bands for the bulk ScN, as displayed in Figure 2f, are quite similar to the MBJGGA-derived ones [19]. One may only notice an increased valence bandwidth in the HSE band structure. The reasonable results for bulk compounds and the lack of any experimental data for systems studied here make the arbitrary selection of α impossible.

The band structure for the 2L ScN is depicted in Figure 2b. The presence of an additional atomic layer in this system led to a doubled number of bands. The valence bandwidth was increased and the localization, as well as the shapes of the additional bands, were slightly different from those in the 1L ScN. These effects were further enhanced in materials with bigger numbers of atomic layers, as shown in Figure 2c–e. Some common trends in the band structures between these systems may be noticed, namely, the valence and conduction bands at the Γ point in the few-layer slabs exhibited relatively high energies with respect to the Fermi energy ($E_{F}$), which resulted in the CBM at the *K* point in the 4L and VBM at the Γ point in the 5L materials. Although the bulk band structure of the ScN was incompletely resembled in the 5L case, one may notice a clear direction of an evolution of the highest valence and lowest conduction bands in the 1L–5L slabs.

**Figure 3:**
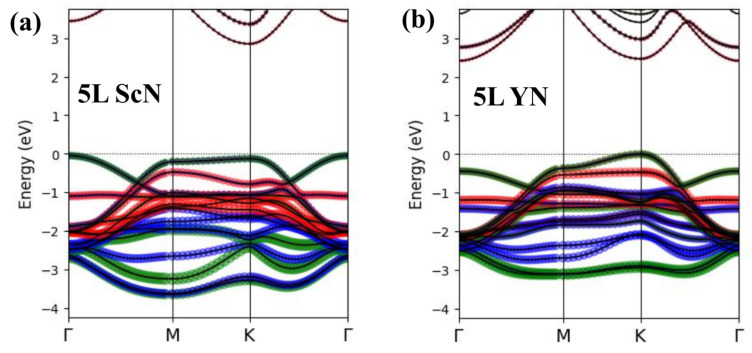
Band character plots for the 5L ScN (**a**) and 5L YN (**b**) systems. The contributions from the first, second, and third atomic layers are marked with red, blue, and green colors, respectively.

The strong changes in the positions and overall shapes of the VBM in the monolayer and bulk *RE*N systems are directly connected with orbital characters of valence bands, as reported in the previous study [19]. The bulk VBM at the Γ point was formed by the $p_{z}$ states, whereas the $p_{x+y}$ states of nitrogen were dominant along the *M*–*K* line. The conduction band region in the *RE* nitrides exhibited the *d*-type character related to the *RE* ions. In the *RE*N monolayer systems, the valence $p_{z}$-type bands were almost flat and located ≈0.7 eV below $E_{F}$, which led to the $p_{x+y}$-type VBM being located at the *M*/*K* point. The $p_{z}$ bands became less flat for the 2L and bigger slabs, which resulted in their presence in the vicinity of $E_{F}$, and the eventual formation of a new VBM at the Γ point. Similar changes in the VBM/CBM positions were less pronounced in few-layer hBN [34]. The complex variation in band alignments in *RE*N slabs requires further discussion. The careful analysis of band characters in the 5L ScN system, depicted in Figure 3a, revealed that the bands came from the surface as rather narrow and located well below the VBM. Although the bands formed by the subsurface atomic layers were mainly located in the region of relatively high binding energy, their contributions to the highest occupied band were crucial for the formation of the VBM in multilayer systems. The presence of every additional atomic layer in hexagonal *RE*N semiconductors led to significant modifications of the VBM of the host material. The bulk-like position and shapes of the valence and conduction bands may therefore be obtained in very big slabs only.

**Figure 4:**
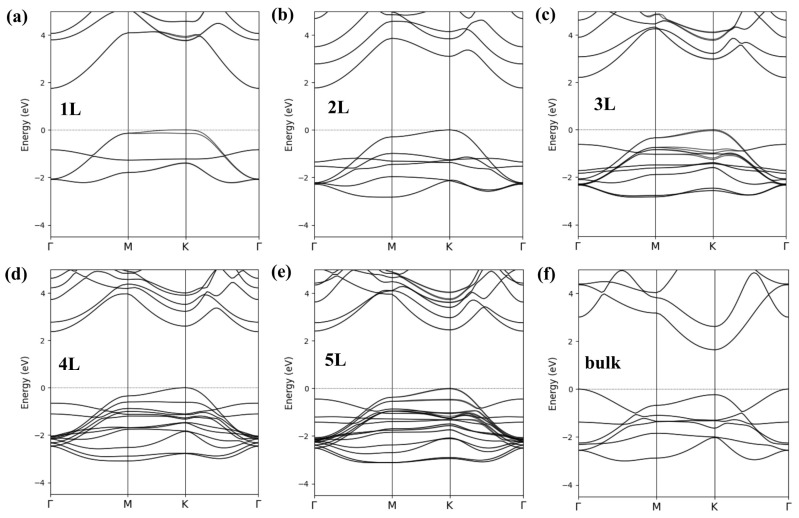
Band structures calculated within the HSE approach for the 1L (**a**), 2L (**b**) 3L (**c**), 4L (**d**), 5L (**e**), and bulk (**f**) hexagonal YN systems.

The band structure for the YN monolayer is presented in Figure 4a. It was generally similar to that of the 1L ScN. The band positions and shapes in the vicinity of the *K* point in the systems studied were connected with an ionic radius of a particular *RE* element. The VBM splittings in band structures, which were present in the *M*–*K*–Γ lines, were connected with the SOC strength in a particular monolayer. This effect was revealed in fully relativistic results for 1L YN and was not present in previous scalar relativistic studies on this system [13]. As one may expect, the effect of the SOC on the band structure of the 1L ScN was rather negligible, whereas the most pronounced band features related to the SOC were reported for 1L LuN [19]. The value of $E_{g}^{K-\Gamma }$ of 1.75 eV predicted for the 1L YN was bigger than the previous MBJGGA-based result (1.36 eV [19]) and $E_{g}^{\Gamma -K}$ of 1.64 eV obtained for the bulk YN. It is worth noting that the variation in $E_{g}$ between the bulk and 1L YNs was weaker than that revealed for the corresponding Sc-based systems.

The VBM of the multilayer YN materials was located at the K point, as depicted in Figure 4b–e. This characteristic shape of the valence bands in the vicinity of $E_{F}$ was similar to that of hBN [34]. The evolution of the valence and conduction bands related to the increasing number of atomic layers was less pronounced in the Y- than Sc-based nitride systems. The bulk-like VBM and CBM may therefore be present in slabs with a very big number of atomic layers. According to the band character plots presented in Figure 3b, the contributions of subsurface ions into the VBM of the 5L YN were dominant. The presence of bands that came from the third atomic layer in a whole valence region of the 5L *RE*N materials is worth emphasizing.

**Figure 5:**
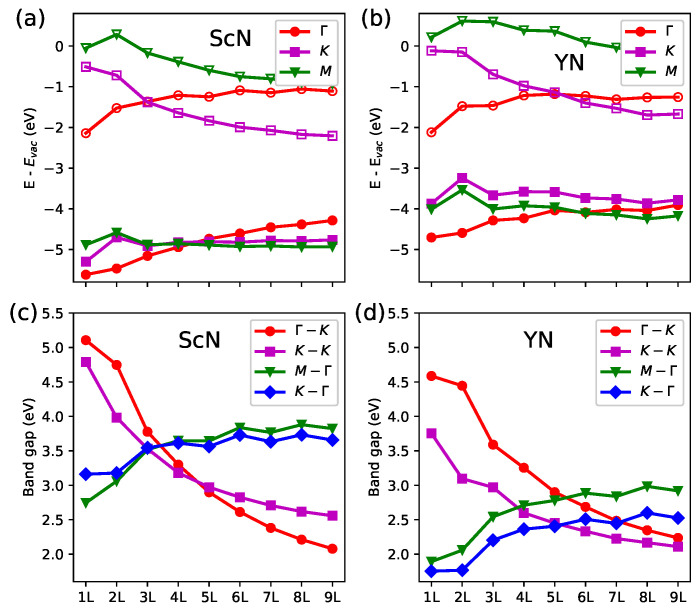
Band energies at selected **k**-points with respect to the vacuum level for the Sc- (**a**) and Y-based (**b**) multilayer nitride systems. The valence and conduction bands are marked with open and full symbols, respectively. The particular band gaps calculated for the Sc- (**c**) and Y-based (**d**) systems.

The positions of the highest occupied and lowest unoccupied bands with respect to the vacuum level in the 1L–9L ScN and YN are displayed in Figure 5a,b. The above-discussed observation of the increased energies of the valence and conduction bands at the Γ point in the big slabs was clearly pronounced. The positions of the valence bands at other **k**-points were almost constant in the 3L–9L ScN or even shifted down in the Y-based systems. These effects led eventually to the VBM located at the Γ point in the 5L ScN, which was also characteristic of the bulk ScN compound. Unfortunately, the fully relativistic HSE calculations of a comparable bulk vacuum level would require exceptionally high computational costs due to a relatively big number of bands and the slow convergence of band structures as a function of the number of atomic layers in the *RE*N slabs. The results obtained for the 1L–9L systems were sufficient for a careful discussion of the general trends present in the band structures. The evolution of the conduction band in the studied materials revealed a pronounced shift into a lower energy region, which resulted in the CBM at the *K* point in slabs bigger than 2L and 4L for the Sc- and Y-based systems.

The band offsets presented in Figure 5a,b indicate a strong variation in possible band alignments in the homo- and heterojunctions of multilayer hexagonal *RE*N semiconductors. The straddling band gaps are expected in systems formed by slabs and bulk ScN, whereas staggered band alignments are possible in heterostructures and between some Y-based slabs. It is worth recalling that the similar dependences of the band position as a function of a number of atomic layers were reported for Mo- and W-based dichalcogenides [20].

Selected band gaps of the ScN systems are presented in Figure 5c. The indirect $E_{g}^{M-\Gamma }$ and $E_{g}^{K-\Gamma }$ characteristic of 1L increased, whereas the relatively wide $E_{g}^{K-K}$ and $E_{g}^{\Gamma -K}$ rapidly decreased with an increasing number of ScN slab layers. The values of $E_{g}^{M-\Gamma }$ and $E_{g}^{K-\Gamma }$ were comparable in the 2L slab; $E_{g}^{M-\Gamma }$, $E_{g}^{K-\Gamma }$, and $E_{g}^{K-K}$ were comparable in the 3L slab; and $E_{g}^{K-K}$ and $E_{g}^{\Gamma -K}$ were comparable in the 4L–5L slabs. These effects resulted in a complex variation in fundamental gaps between the ScN-based few-layer systems. Although the $E_{g}$ was relatively wide in the 1L case, it could be even wider in the 2L–5L materials and become narrower in bigger slabs. In the case of the Y-based slabs, the variation in $E_{g}$ was smaller. As presented in Figure 5d, the interplay of $E_{g}^{K-\Gamma }$ and $E_{g}^{K-K}$ was crucial for the band gaps of the 1L–9L YN materials. One may expect that the bulk-like $E_{g}^{\Gamma -K}$ became dominant in systems that are much bigger than 9L. The strong increase in the band gaps of the monolayer with respect to the bulk *RE*N systems has also been reported in previous full-potential MBJGGA studies [19]. On the one hand, the electronic structure of the 2D nitride materials may be somehow exceptional. On the other hand, the results of PBE and GW calculations for multilayer transition metal dichalcogenides also indicate a rather slow convergence of band gaps with respect to those of bulk compounds [20]. Even a less pronounced variation in the $E_{g}$ than the HSE-derived results discussed here may be considered as a promising feature for some applications of hexagonal *RE*N semiconductors in optoelectronics, e.g., heterostructures or superlattices with well-tuned band gaps.

**Figure 6:**
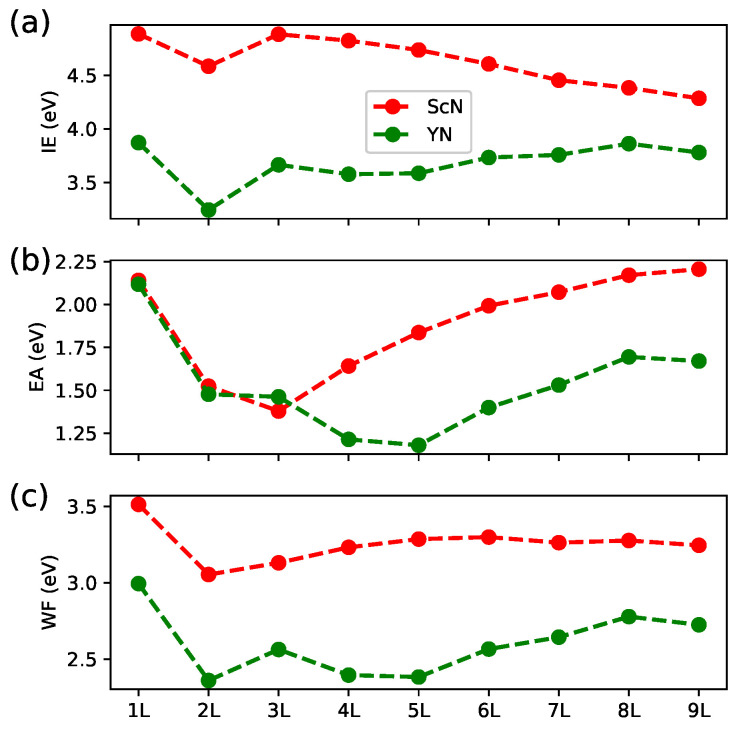
Ionization energy (IE) (**a**), electron affinity (EA) (**b**), and work function (WF) (**c**) calculated for multilayer ScN and YN materials.

The values of ionization energy (IE), electron affinity (EA), and work function (WF) calculated for the multilayer ScN and YN are presented in Figure 6. The maximum values of the IE were obtained for the monolayers. A decrease in the IE was observed in the 3L–9L ScN, whereas in the corresponding YN slabs, a slight increase in the IE was revealed. Other monolayer materials exhibited generally stronger IEs, e.g., hBN and WSe_2_ with an IE above 6 eV [20,34]. The variation in the EA was more complex and depended on the *RE* ion present in a particular material. As depicted in Figure 6b, the highest values of the EA were obtained for the 1L systems. The reduced IE of 1.25 eV expected in some multilayer YN materials was bigger than that reported for the hBN (0.86 eV [34]) and lower than those of the TMDC (above 3 eV [20]). Except for the increased WF in 1L, the values of WF were rather comparable between the 2L–9L materials studied here. The WF of the ScN slabs was significantly bigger than that of the Y-bearing systems. This was, however, low compared with the WF of 5.74 eV reported for 1L BN [35]. The wurtzite GaN and InN exhibited a WF of 4.1 eV [36,37], whereas a WF of 4–5 eV was predicted for Mo- and W-based dichalcogenides [20]. The low values of WF in the *RE*N systems were not surprising considering the previous findings and careful discussion reported for transition metal nitrides [38]. Even lower values of the WF (2.16 eV for HfN) were obtained with DFT calculations for members of this family of compounds. Further efforts to reduce the WF in 2D hexagonal YN might be an interesting area for theoretical and experimental research considering potential applications of this system as an electron emission material.

## 4. Conclusions

The structural parameters of 2D hexagonal Sc- and Y-based nitride materials were expected to exhibit pronounced changes related to the number of atomic layers in a particular system. A significant reduction in the in-plane lattice parameter obtained for slabs with a small number of atomic layers was connected with a decrease in the out-of-plane distance between the first and second atomic layers, and further balanced by a slight relaxation of the position of the third atomic layers of *RE*N materials. The bulk-like distance between the atomic layers was expected in systems bigger than 5L–6L. A complex evolution of band structures obtained with a hybrid exchange-correlation functional for the 1L–9L ScN and YN did not lead to the corresponding bulk-like electronic structures of these materials. A characteristic interplay between the positions of valence and conduction bands with respect to the vacuum level were found among the systems studied. The indirect band gaps, $E_{g}^{M-\Gamma }$ and $E_{g}^{K-\Gamma }$, were dominant in the small slabs of Sc- and Y-based materials, respectively. The $E_{g}^{\Gamma -K}$ energy gap, which is characteristic of bulk systems, was expected to be a fundamental band gap in the ScN slabs bigger than 4L and YN slabs bigger than 9L. A reduction in the relatively low ionization energy, electron affinity, and work function predicted for few-layer YN might lead to the discovery of a novel material for electron emission. The diverse electronic structure of multilayer systems presented in this work encourages further experimental efforts in the synthesis of hexagonal *RE* nitride semiconductors.

## Figures and Tables

**Table 1:** Hexagonal lattice parameters *a*, *c*, and *c/a* ratios calculated in this work and available literature data for hexagonal bulk and monolayer ScN, YN, and LuN.

System	*a* (Å)	*c* (Å)	c/a
ScN bulk:			
GGA	3.715	4.475	1.205
D2	3.646	4.454	1.221
D3	3.654	4.418	1.209
LDA [19]	3.662	4.416	1.206
YN bulk:			
GGA	4.011	4.880	1.217
D2	3.924	4.856	1.237
D3	3.943	4.815	1.221
LDA [19]	3.965	4.829	1.218
LuN bulk LDA [19]	3.849	4.661	1.211
ScN monolayer:			
GGA	3.518	-	-
GGA [8]	3.510	-	-
GGA [12]	3.520	-	-
LDA [19]	3.460	-	-
YN monolayer:			
GGA	3.795	-	-
LDA [19]	3.758	-	-
GGA+*U* [13]	4.008	-	-
LuN monolayer LDA [19]	3.652	-	-

## Data Availability

The raw data supporting the conclusions of this article will be made available by the authors on request.

## References

[1] Caldwell J.D., Aharonovich I., Cassabois G., Edgar J.H., Gil B., Basov D.N. (2019). Photonics with hexagonal boron nitride. Nat. Rev. Mater..

[2] Farrer N., Bellaiche I. (2002). Properties of hexagonal ScN versus wurtzite GaN and InN. Phys. Rev. B.

[3] Constantin C., Al-Brithen H., Haider M.B., Ingram D., Smith A.R. (2004). ScGaN alloy growth by molecular beam epitaxy: Evidence for a metastable layered hexagonal phase. Phys. Rev. B.

[4] Winiarski M.J., Kowalska D. (2019). Electronic structure of REN (RE = Sc, Y, La, and Lu) semiconductors by MBJLDA calculations. Mater. Res. Express..

[5] Winiarski M.J., Kowalska D.A. (2020). Crystal structure of ternary alloys of group III and rare earth nitrides by ab initio calculations. Sci. Rep..

[6] Winiarski M.J. (2021). Electronic Structure of Ternary Alloys of Group III and Rare Earth Nitrides. Materials.

[7] Tamleh S., Rezaei G., Jalilian J. (2018). Stress and strain effects on the electronic structure and optical properties of ScN monolayer. Phys. Lett. A.

[8] Tamleh S., Rezaei G., Vaseghi B., Jalilian J. (2020). Electronic structure and optical properties of two-dimensional tetragonal and hexagonal ScN monolayers: Impact of strain. J. Phys. Chem. Solids.

[9] Liang D., Jing T., Deng M., Cai S. (2021). Two-dimensional ScN with high carrier mobility and unexpected mechanical properties. Nanotechnology.

[10] Fakhrabad D.V., Yeganeh M. (2022). Investigation of the effect of lattice thermal conductivity on the thermoelectric performance of ScN monolayer. Mater. Sci. Semicond. Process..

[11] Yeganeh M., Fakhrabad D.V. (2022). Piezoelectric properties in hydrofluorination surface-engineered two-dimensional ScN. Micro Nanostruct..

[12] On V.V., Guerrero-Sanchez J., Hoat D.M. (2024). Modifying the electronic and magnetic properties of the scandium nitride semiconductor monolayer via vacancies and doping. Phys. Chem. Chem. Phys..

[13] Zheng K., Yang X., Cui H., Yang Q., Ye H., Xiong D., Ingebrandt S., Chen X. (2018). Intriguing electronic insensitivity and high carrier mobility in monolayer hexagonal YN. J. Mater. Chem. C.

[14] Gall D., Stadele M., Jarrendahl K., Petrov I., Desjardins P., Haasch R.T., Lee T.-Y., Greene J.E. (2001). Electronic structure of ScN determined using optical spectroscopy, photoemission, and ab initio calculations. Phys. Rev. B.

[15] Qteish A., Rinke P., Scheffler M., Neugebauer J. (2006). Exact-exchange-based quasiparticle energy calculations for the band gap, effective masses, and deformation potentials of ScN. Phys. Rev. B.

[16] Ramirez-Montes L., Lopez-Perez W., Gonzalez-Garcia A., Gonzalez-Hernandez R. (2016). Structural, optoelectronic, and thermodynamic properties of Y_*x*_Al_1−*x*_N semiconducting alloys. J. Mater. Sci..

[17] Cherchab Y., Azzouz M., Gonzalez-Hernandez R., Talbi K. (2014). First-principles prediction of the structural and electronic properties of Ga_*x*_Y_1−*x*_N compounds. Comput. Mater. Sci..

[18] Singh S.K., Verma U.P. (2015). Investigation of high pressure phase transition and electronic properties of Lutetium Nitride. J. Phys. Conf. Ser..

[19] Winiarski M.J., Kowalska D.A. (2022). Electronic structure of hexagonal REN (RE = Sc, Y, and Lu) materials. Mater. Chem. Phys..

[20] Kim H., Choi H.J. (2021). Thickness dependence of work function, ionization energy, and electron affinity of Mo and W dichalcogenides from DFT and GW calculations. Phys. Rev. B.

[21] Krukau A.V., Vydrov O.A., Izmaylov A.F., Scuseria G.E. (2006). Influence of the exchange screening parameter on the performance of screened hybrid functionals. J. Chem. Phys..

[22] Kresse G., Hafner J. (1993). Ab initio molecular dynamics for liquid metals. Phys. Rev. B.

[23] Kresse G., Furthmüller J. (1996). Efficient iterative schemes for ab initio total-energy calculations using a plane-wave basis set. Phys. Rev. B.

[24] Kresse G., Joubert D. (1999). From ultrasoft pseudopotentials to the projector augmented-wave method. Phys. Rev. B.

[25] Perdew J.P., Burke K., Ernzerhof M. (1996). Generalized Gradient Approximation Made Simple. Phys. Rev. Lett..

[26] Grimme S. (2006). Semiempirical GGA-type density functional constructed with a long-range dispersion correction. J. Comput. Chem..

[27] Grimme S., Antony J., Ehrlich S., Krieg H. (2010). A consistent and accurate ab initio parametrization of density functional dispersion correction (DFT-D) for the 94 elements H-Pu. J. Chem. Phys..

[28] Lin S., Ye X., Johnson R.S., Guo H. (2013). First-Principles Investigations of Metal (Cu, Ag, Au, Pt, Rh, Pd, Fe, Co, and Ir) Doped Hexagonal Boron Nitride Nanosheets: Stability and Catalysis of CO Oxidation. J. Phys. Chem. C.

[29] Keum D.H., Cho S., Kim J.H., Choe D.-H., Sung H.-J., Kan M., Kang H., Hwang J.-Y., Kim S.W., Yang H. (2015). Bandgap opening in few-layered monoclinic MoTe_2_. Nat. Phys..

[30] Shi J., Zeng Q., Chen Y., Niu L., Liu F., Yu T., Suenaga K., Liu X., Lin J. (2018). InSe monolayer: Synthesis, structure and ultra-high second-harmonic generation. 2D Mater..

[31] Okada M., Sawazaki T., Watanabe K., Taniguch T., Hibino H., Shinohara H., Kitaura R. (2014). Direct Chemical Vapor Deposition Growth of WS_2_ Atomic Layers on Hexagonal Boron Nitride. ACS Nano.

[32] Islam R., Ghosh B., Autieri C., Chowdhury S., Bansil A., Agarwal A., Singh B. (2021). Tunable spin polarization and electronic structure of bottom-up synthesized MoSi_2_N_4_ materials. Phys. Rev. B.

[33] Ota Y. (2018). Band alignments of graphene-like III-nitride semiconductors. Solid State Commun..

[34] Wickramaratne D., Weston L., van de Walle C.G. (2018). Monolayer to bulk properties of hexagonal boron nitride. J. Phys. Chem. C.

[35] Thomas S., Manju M.S., Ajith K.M., Lee S.U., Asle Zaeem M. (2020). Strain-induced work function in h-BN and BCN monolayers. Phys. E.

[36] Schultz T., Schlesinger R., Niederhausen J., Henneberger F., Sadofev S., Blumstengel S., Vollmer A., Bussolotti F., Yang J.-P., Kera S. (2016). Tuning the work function of GaN with organic molecular acceptors. Phys. Rev. B.

[37] Himmerlich M., Krischok S., Lebedev V., Ambacher O., Schaefer J.A. (2007). Morphology and surface electronic structure of MBE grown InN. J. Cryst. Growth.

[38] Ma T., Jacobs R., Booske J., Morgan D. (2021). Work Function Trends and New Low-Work-Function Boride and Nitride Materials for Electron Emission Applications. J. Phys. Chem. C.

